# Supplementary material for: Association of serum Metrnl levels and high-density lipoprotein cholesterol in patients with type 2 diabetes mellitus: a cross-sectional study
Source: PeerJ. 2024 Oct 14;12:e18264. doi: 10.7717/peerj.18264 (PMC11485050; doi:10.7717/peerj.18264)
Supplement: Supplemental Information 1 — Model1: unadjusted Model2: adjusted for sex, age Model3: adjusted for sex, age, HDL-C HDL-C: high-density lipoprotein cholesterol [file peerj-12-18264-s001.docx]

**Supplementary Table 1: The relationship between serum adiponectin level and Metrnl (Multivariate ordinal logistic regression)**

|  | OR (95% CI) | P |
| --- | --- | --- |
| Model1 | 1.09(0.02-0.15) | 0 .011 |
| Model2 | 1.10 (0.02-1.62) | 0 .011 |
| Model3 | 1.10 (0.02-0.17) | 0 .020 |

Model1: unadjusted

Model2: adjusted for sex, age

Model3: adjusted for sex, age, HDL-C

HDL-C : high-density lipoprotein cholesterol
