# Supplementary material for: Association of serum Metrnl levels and high-density lipoprotein cholesterol in patients with type 2 diabetes mellitus: a cross-sectional study
Source: PeerJ. 2024 Oct 14;12:e18264. doi: 10.7717/peerj.18264 (PMC11485050; doi:10.7717/peerj.18264)
Supplement: Supplemental Information 5 [file peerj-12-18264-s005.docx]

STROBE Statement—checklist of items that should be included in reports of observational studies

|  | **Item No.** | **Recommendation** | **Page No.** | **Relevant text from manuscript** |
| --- | --- | --- | --- | --- |
| **Title and abstract** | 1 | (*a*) Indicate the study’s design with a commonly used term in the title or the abstract | 1 | Association of serum metrnl levels and high-density lipoprotein cholesterol in patients with type 2 diabetes mellitus: a cross-sectional study |
|  |  | (*b*) Provide in the abstract an informative and balanced summary of what was done and what was found | 2 | Abstract |
| **Introduction** | | | | |
| Background/rationale | 2 | Explain the scientific background and rationale for the investigation being reported | 4 | Metrnl, an adipokine that was first characterized in 2014 |
| Objectives | 3 | State specific objectives, including any prespecified hypotheses | 5 | Consequently, the primary objective of this study was to examine the potential correlation between serum Metrnl concentrations and HDL-C levels among patients diagnosed with T2DM |
| **Methods** | | | | |
| Study design | 4 | Present key elements of study design early in the paper | 5 | Study Population |
| Setting | 5 | Describe the setting, locations, and relevant dates, including periods of recruitment, exposure, follow-up, and data collection | 5 | Study Population |
| Participants | 6 | (*a*) *Cohort study*—Give the eligibility criteria, and the sources and methods of selection of participants. Describe methods of follow-up  *Case-control study*—Give the eligibility criteria, and the sources and methods of case ascertainment and control selection. Give the rationale for the choice of cases and controls *Cross-sectional study*—Give the eligibility criteria, and the sources and methods of selection of participants | 5 | Between June 2022 and December 2022, a group of participants with type 2 diabetes mellitus were recruited from the… |
|  |  | (*b*) *Cohort study*—For matched studies, give matching criteria and number of exposed and unexposed  *Case-control study*—For matched studies, give matching criteria and the number of controls per case |  |  |
| Variables | 7 | Clearly define all outcomes, exposures, predictors, potential confounders, and effect modifiers. Give diagnostic criteria, if applicable | 5 | The diagnosis of diabetes was made in accordance with the standards set by the World Health Organization in 2009 |
| Data sources/ measurement | 8* | For each variable of interest, give sources of data and details of methods of assessment (measurement). Describe comparability of assessment methods if there is more than one group | *5-6* | Anthropometric and biochemical assessment, Statistical Analysis |
| Bias | 9 | Describe any efforts to address potential sources of bias | 5 | Every participant provided informed written consent, and the research procedure was ethically approved by Shanghai East Hospital's respective ethics committees. |
| Study size | 10 | Explain how the study size was arrived at | 5 | Ultimately, a total of 80 participants were included in the final evaluation |
| Quantitative variables | 11 | Explain how quantitative variables were handled in the analyses. If applicable, describe which groupings were chosen and why | 6 | **Grouping** |
| Statistical  methods | 12 | (a) Describe all statistical methods, including those used to control for confounding | 6 | Statistical Analysis |
|  |  | (b) Describe any methods used to examine subgroups and interactions |  |  |
|  |  | (c) Explain how missing data were addressed |  |  |
|  |  | (d) Cohort study—If applicable, explain how loss to follow-up was addressed  Case-control study—If applicable, explain how matching of cases and controls was addressed  Cross-sectional study—If applicable, describe analytical methods taking account of sampling  strategy |  |  |
|  |  | (e) Describe any sensitivity analyses |  |  |
| **Results** | | | | |
| Participants | 13* | (a) Report numbers of individuals at each stage of study—eg numbers potentially eligible, examined  for eligibility, confirmed eligible, included in the study, completing follow-up, and analysed | 7 | Based on their HDL-C levels, participants were split into two groups (Group1: <1.04; Group2: ≥1.04) (Table1). |
|  |  | (b) Give reasons for non-participation at each stage |  |  |
|  |  | (c) Consider use of a flow diagram |  |  |
| Descriptive data | 14* | (a) Give characteristics of study participants (eg demographic, clinical, social) and information on  exposures and potential confounders | 7 | Age, gender, and the duration of illness do not differ significantly in statistics between the two groups. The group with decreased HDL-C levels had higher levels of FPG, 2 h PG, HOMA-IR, HbA1c, and TG (P < 0.05). Patients with greater HDL-C groups had higher serum Metrnl levels (P = 0.006) (Figure 1). |
|  |  | (b) Indicate number of participants with missing data for each variable of interest |  |  |
|  |  | (c) Cohort study—Summarise follow-up time (eg, average and total amount) |  |  |
| Outcome data | 15* | Cohort study—Report numbers of outcome events or summary measures over time |  |  |
|  |  | Case-control study—Report numbers in each exposure category, or summary measures of exposure |  |  |
|  |  | Cross-sectional study—Report numbers of outcome events or summary measures | 7 | Based on their HDL-C levels, participants were split into two groups (Group1: <1.04; Group2: ≥1.04) (Table1). |
| Main results | 16 | (a) Give unadjusted estimates and, if applicable, confounder-adjusted estimates and their precision  (eg, 95% confidence interval). Make clear which confounders were adjusted for and why they were  included | 7-8 | A noteworthy inverse relationship was found in an unadjusted logistic regression model (Model 1), (OR = 0.250, 95% CI: 0.080-0.781, P = 0.017), between lower serum Metrnl concentrations and higher HDL-C relative to the highest tertile. To put it another way, it shows that serum Metrnl concentrations and HDL-C levels are positively correlated. |
|  |  | (b) Report category boundaries when continuous variables were categorized | 8 | each participant was separated into three groups (Table 4) based on the tertiles of their Metrnl levels: 3.290 ng/mL for Tertile 1; 31.290–37.776 ng/mL for Tertile 2; and 37.776 ng/mL for Tertile 3. |
|  |  | (c) If relevant, consider translating estimates of relative risk into absolute risk for a meaningful time period |  |  |
| Other analyses | 17 | Report other analyses done—eg analyses of subgroups and interactions, and sensitivity analyses |  |  |
| **Discussion** | | | | |
| Key results | 18 | Summarise key results with reference to study objectives | 9 | In T2DM patients, our research revealed a beneficial correlation between blood concentrations of Metrnl and HDL-C, which may provide an objective for the prognosis and prevention of diabetic cardiovascular disease. |
| Limitations | 19 | Discuss limitations of the study, taking into account sources of potential bias or imprecision. Discuss both direction and magnitude of any potential bias | 13 | There were several inherent flaws in the design of the current study. Firstly, it was a cross-sectional study without follow-up, which means that more work is needed to consolidate the findings. |
| Interpretation | 20 | Give a cautious overall interpretation of results considering objectives, limitations, multiplicity of analyses, results from similar studies, and other relevant evidence | 11-12 | In previous studies, Metrnl has been demonstrated to inhibit steatosis, promote lipid metabolism, and ultimately improve insulin resistance in adipocyte through PPARγ pathway via an autocrine or paracrine mechanism |
| Generalisability | 21 | Discuss the generalisability (external validity) of the study results | 12 | We further compared the HDL-C levels with different Metrnl levels in T2DM patients. Similarly, it showed that individuals with the lowest Metrnl concentrations had lower HDL-C levels. |
| **Other information** | | | | |
| Funding | 22 | Give the source of funding and the role of the funders for the present study and, if applicable, for the original study on which the present article is based | 13-14 | This work was supported by Academic Leaders Training Program of Pudong Health Bureau of Shanghai, NO. PWRd2023-03; Clinical Research Fund of Shanghai Municipal Commission of Health, NO. 202040136; National Natural Science Foundation of China, NO. 82070842; and Jiangxi Health Commission Science and Technology Plan Project, NO.202212838, 202212852. |

Continued on next page

*Give information separately for cases and controls in case-control studies and, if applicable, for exposed and unexposed groups in cohort and cross-sectional studies.

**Note:** An Explanation and Elaboration article discusses each checklist item and gives methodological background and published examples of transparent reporting. The STROBE checklist is best used in conjunction with this article (freely available on the Web sites of PLoS Medicine at http://www.plosmedicine.org/, Annals of Internal Medicine at http://www.annals.org/, and Epidemiology at http://www.epidem.com/). Information on the STROBE Initiative is available at www.strobe-statement.org.
