# Supplementary material for: Association of serum Metrnl levels and high-density lipoprotein cholesterol in patients with type 2 diabetes mellitus: a cross-sectional study
Source: PeerJ. 2024 Oct 14;12:e18264. doi: 10.7717/peerj.18264 (PMC11485050; doi:10.7717/peerj.18264)
Supplement: Supplemental Information 6 [file peerj-12-18264-s006.docx]

Manuscript #97828

Title: Association of serum metrnl levels and high-density lipoprotein cholesterol in patients with type 2 diabetes mellitus: a cross-sectional study

Subject: Author change request for the manuscript titled “Association of serum metrnl levels and high-density lipoprotein cholesterol in patients with type 2 diabetes mellitus: a cross-sectional study”（Manuscript ID#97828）

Dear editor：

We would like to add Bo Feng as co- correspondence author because professor Feng contributed to the study design, protocol development, ethical approval, study supervision and critical revision of the manuscript. In fact, we added Feng Bo's name to the author list of the manuscript before the first decision, but due to our negligence, he was omitted in the submission system. So, he was added when we revised it, and all authors agree to the changes. We must declare that he did participate in the study and should be listed as a co-author for his contributions. Dr Feng is an experienced clinician who has directed many clinical trials. We believe that his guidance improved the study a lot. Dr Feng’s contributions was clearly tracked in the updated tracked changes manuscript.

We apologize for the inconvenience and kindly hope your approval for this authorship modification. Please let us know if you need any additional information or clarification, or if there are any specific steps we should take to update the author list.

Thank you for your time and consideration.

Sincerely,

Chenxia Zhou

Jun Song, Department of Endocrinology, East Hospital, Tongji University School of Medicine, 150 Jimo Road, Shanghai, 200120, People’s Republic of China, Tel/Fax +86 021-38804518, Email: songjuntj@tongji.edu.cn;
